# Microarray-based detection and expression analysis of new genes associated with drug resistance in ovarian cancer cell lines

**DOI:** 10.18632/oncotarget.18278

**Published:** 2017-05-29

**Authors:** Radosław Januchowski, Karolina Sterzyńska, Piotr Zawierucha, Marcin Ruciński, Monika Świerczewska, Małgorzata Partyka, Katarzyna Bednarek-Rajewska, Maciej Brązert, Michał Nowicki, Maciej Zabel, Andrzej Klejewski

**Affiliations:** ^1^ Department of Histology and Embryology, Poznań University of Medical Sciences, Poznań, 60-781, Poland; ^2^ Department of Anatomy, Poznań University of Medical Sciences, Poznań, 60-781, Poland; ^3^ Department of Clinical Pathomorphology, Poznań University of Medical Sciences, Poznań, 60-355, Poland; ^4^ Division of Infertility and Reproductive Endocrinology, Department of Gynecology, Obstetrics and Gynecological Oncology, Poznań University of Medical Sciences, Poznań, 60-535, Poland; ^5^ Department of Histology and Embryology, Wrocław Medical University, Wrocław, 50-368, Poland; ^6^ Department of Nursing, Poznań University of Medical Sciences, Poznań, 60-179, Poland; ^7^ Departament of Obstetrics and Womens Dieseases, Poznań University of Medical Sciences, Poznań, 60-535, Poland

**Keywords:** ovarian cancer, drug resistance, cDNA microarray, new genes

## Abstract

**Purpose:**

The present study is to discover a new genes associated with drug resistance development in ovarian cancer.

**Methods:**

We used microarray analysis to determine alterations in the level of expression of genes in cisplatin- (CisPt), doxorubicin- (Dox), topotecan- (Top), and paclitaxel- (Pac) resistant variants of W1 and A2780 ovarian cancer cell lines. Immunohistochemistry assay was used to determine protein expression in ovarian cancer patients.

**Results:**

We observed alterations in the expression of 22 genes that were common to all three cell lines that were resistant to the same cytostatic drug. The level of expression of 13 genes was upregulated and that of nine genes was downregulated. In the CisPt-resistant cell line, we observed downregulated expression of ABCC6, BST2, ERAP2 and MCTP1; in the Pac-resistant cell line, we observe upregulated expression of ABCB1, EPHA7 and RUNDC3B and downregulated expression of LIPG, MCTP1, NSBP1, PCDH9, PTPRK and SEMA3A. The expression levels of three genes, ABCB1, ABCB4 and IFI16, were upregulated in the Dox-resistant cell lines. In the Top-resistant cell lines, we observed increased expression levels of ABCG2, HERC5, IFIH1, MYOT, S100A3, SAMD4A, SPP1 and TGFBI and decreased expression levels of MCTP1 and PTPRK. The expression of EPHA7, IFI16, SPP1 and TGFBI was confirmed at protein level in analyzed ovarian cancer patients..

**Conclusions:**

The expression profiles of the investigated cell lines indicated that new candidate genes are related to the development of resistance to the cytostatic drugs that are used in first- and second-line chemotherapy of ovarian cancer.

## INTRODUCTION

Ovarian cancer is one of the most lethal gynecological malignancies. Most patients are diagnosed with an advanced disease and have a poor prognosis [1]. At the beginning of treatment, ovarian cancer is one of the most treatable solid tumours. However, during treatment, ovarian cancer cells may develop drug resistance, causing further treatments to be ineffective in most cases [2].

The most significant mechanism underlying the resistance to cytostatic drugs is their active removal from cancer cells by drug transporters of the ABC family [3]. These transporters pump cytostatic drugs from cancer cells using energy derived from ATP hydrolysis [4]. The most important ABC transporters are glycoprotein P (P-gp), which is encoded by the ABCB1 (multidrug resistance protein 1-MDR1) gene [5], and the breast-cancer resistance protein BCRP, which is encoded by the ABCG2 gene [6]. Other mechanisms underlying cytostatic-drug resistance include the following: inactivating the drugs using detoxification enzymes, inactivating the drugs through metallothionein or glutathione binding, repairing damaged DNA, developing point mutations in the genes that encode proteins that bind cytostatic drugs, and increasing the activity of anti-apoptotic or pro-survival pathways as well as disrupting apoptotic signaling pathways [7].

The first-line chemotherapy for advanced ovarian cancer always involves a platinum-based drug [carboplatin or cisplatin (CisPt)] and a taxane [paclitaxel (Pac) or docetaxel] [8]. The second-line chemotherapy in the case of platinum-sensitive disease generally includes platinum-containing compounds and taxane [9]. In the case of platinum-resistant disease, other cytostatic drugs, such as liposomal doxorubicin (Dox), topotecan (Top) and gemcitabine, are used [10, 11].

CisPt is the most frequently used antitumor agent and the most important drug used in ovarian cancer chemotherapy. This drug reacts with the nitrogen atoms of DNA and preferentially reacts with the N-7 atom of deoxyguanylic acid. This process results in intrastrand and interstrand DNA cross-linking, consequently inhibiting DNA synthesis and transcription [12]. Resistance to CisPt can result from the following events: decreased drug uptake [12]; increased reflux by drug transporters of the ABC family such as MRP2 [13]; and increased drug inactivation by sulfhydryl-containing molecules, such as glutathione [14] and metallothioneins [15]. Another important mechanism underlying CisPt resistance is the repair of damaged DNA via DNA repair systems [16]. The role of DNA damage response (DDR) is to protect against genomic instability and therefore prevent oncogenesis. However, it plays a dual role, not only in cancer prevention but also in anticancer therapy. The inhibition of some DDR pathways sensitize the therapy to chemotherapeutic drugs and therefore abrogate the chemoresistance. [17].

The second most important drug for ovarian cancer treatment is Pac. Pac belongs to the family of antimitotic anticancer agents and blocks mitosis through stabilizing microtubules, leading to the blockage of cell division and thus, cell survival [18]. The most important mechanism underlying cancer-cell resistance to Pac is mediated by MDR proteins such as P-gp [19, 20]. However, other drug –transporters, such as ABCB4 (MDR3), can also play a role in Pac resistance [21]. The other most important mechanisms underlying Pac resistance are the development of tubulin mutations [22] and the expression of the less common tubulin isotypes [23].

The most important drugs used in the second-line chemotherapy of ovarian cancer are Dox and Top. Top and Dox are inhibitors of DNA topoisomerase I and II, respectively. Topoisomerases are enzymes that regulate the overwinding or underwinding of the DNA helix [24]. Topoisomerase I acts through scission of the DNA backbone of one strand and mainly affects the transcription and replication complexes [25]. In contrast, topoisomerase II acts through scission of the DNA backbone of two strands and acts mainly after replication has occurred [26]. Inhibiting DNA topoisomerase activities using poisons such as Dox and Top causes the formation of irreversible covalent cross-links between the topoisomerase and DNA, leading to DNA breakage and consequently, to cell death. Additionally, Dox is a planar compound that intercalates into DNA, thereby block transcription and replication. The main mechanism underlying the resistance to Dox and Top is the expression of specific drug transporters; Dox and Top are actively removed from cancer cells by P-gp and BCRP, respectively [6, 27]. The most common mechanism underlying the resistance to topoisomerase inhibitors is the development of mutations that make these enzymes less sensitive to their inhibitors [28].

However, it sometimes is difficult to explain the development of drug resistance in cancer cells based on the patterns of expression of the genes that are known to be involved in drug resistance, which indicates that yet-unknown genes and mechanisms are also involved in drug resistance.

In this study, we compared the gene-expression profiles of ovarian cancer cell lines resistant to the cytostatic drugs used in first-line chemotherapy, CisPt and Pac, and those used in second-line chemotherapy, Dox and Top. Drug-resistant cell lines were derived from primary (W1) or established (A2780) ovarian cancer cell lines. Alterations in the levels of gene expression of the drug-resistant cell lines were determined using oligonucleotide microarrays.

## RESULTS

### Gene-chip scanning and preliminary analysis

The quality of all of the GeneChip expression data was within the “good sample” limits according to the values for the parameters evaluated in the analysis of the preliminary data, such as the background and noise averages, percentage of present calls, presence of internal hybridization controls as increasing signals, presence of poly-A controls as decreasing signals and the GAPDH to β-actin 3′/5′ signal ratios.

### Data analysis, gene lists and evaluations

The genes that were associated with Cis, Dox, Pac or Top resistance were selected. The contribution to drug resistance of the genes for which the expression level was significantly changed in the drug-resistant cells relative to that in their drug-sensitive counterparts by greater than 5-fold and less than 0.2-fold (up-/down-regulation of more than/less than 5 and -5, respectively) was evaluated. Genes with expression levels of between 5- and 0.2-fold those of the controls were considered ‘not significant (NS)' when the gene lists were constructed.

**Figure 1:**
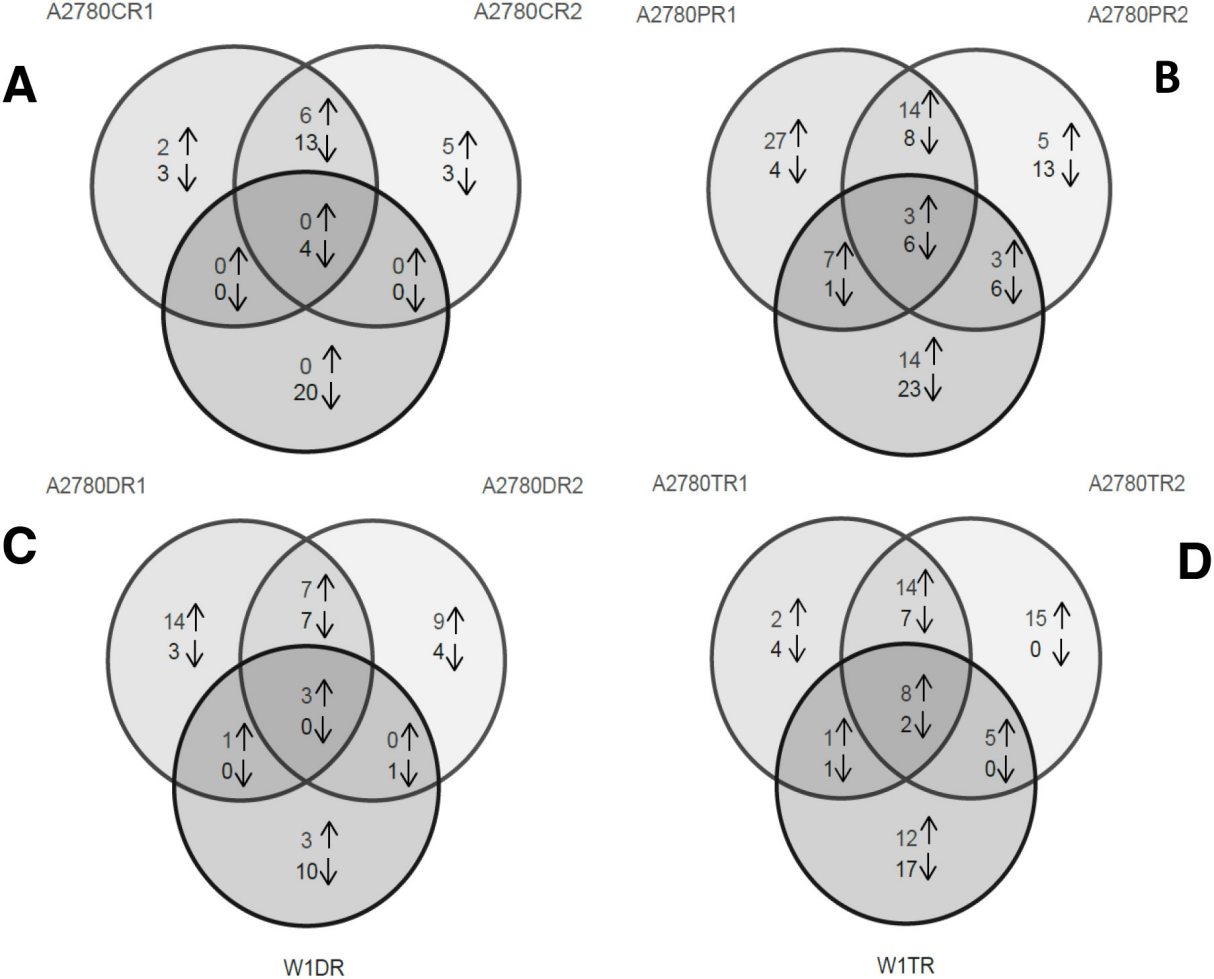
Genes that were overexpressed and underexpressed in the three pairs of cell lines resistant to Cis **(A)**, Pac **(B)**, Dox **(C)** and Top **(D)**. The genes that were overexpressed/underexpressed in more than one cell line are indicated in the overlapping regions of the circles.

Analysis of the gene expression of ovarian cancer cell lines resistant to each cytostatic drug provided information about the response of the cancer cells to the different cytostatic drugs during treatment. Figure 1 is a Venn diagram summarizing the number of transcripts that were overexpressed and underexpressed in each pair of cell lines and the relationship of these transcriptional changes to those of the other cell-line pairs.

In addition to the four genes that were underexpressed in all three of the Cis-resistant cell lines, six genes were overexpressed and 13 genes were underexpressed in both the A2780CR1 and A2780CR2 cell lines.

In addition to the three genes that were overexpressed and the six genes that were underexpressed in all three of the Pac-resistant cell lines, 14 genes were overexpressed and eight genes were underexpressed in both the A2780PR1 and A2780PR2 cell lines. Seven genes were overexpressed and one gene was underexpressed in both the A2780PR1 and W1PR cell lines and three genes were overexpressed and six genes were underexpressed in both the A2780PR2 and W1PR cell lines.

The expression of only three genes was changed (overexpressed) in all three of the Dox-resistant cell lines. The same seven genes were overexpressed and other set of the same seven genes were underexpressed in the A2780DR1 and A2780DR2 cell lines. One gene was overexpressed in both the A2780DR1 and W1DR cell lines and one gene was underexpressed in both the A2780DR2 and W1DR cell lines.

In all three of the Top-resistant cell lines. we observed the upregulated expression of eight genes and the downregulated expression of two genes. Upregulated expression of 14 genes and downregulated expression of seven genes was observed in both the A2780TR1 and A2780TR2 cell lines. One gene was overexpressed and one gene was underexpressed in both the A2780TR1 and W1TR cell lines, and the expression of five genes was upregulated in both the A2780TR2 and W1TR cell lines.

**Figure 2:**
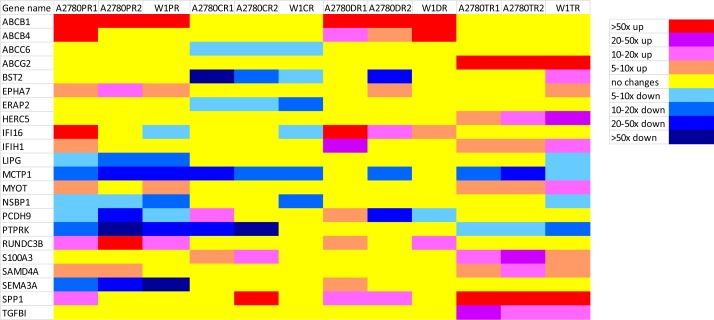
Expression ratios of cytostatic-drug resistance related genes in the drug-resistant sublines

For more detailed analysis, we selected genes with expression that was up/down-regulated more than/less than 5 and -5, respectively, in three cell lines that were resistant to the same cytostatic drug. Table 1 and Figure 2 summarize our results.

**Table 1:** List of the genes and the fold changes in the expression of genes encoding cytostatic-drug resistance-related proteins in the drug-resistant sublines. NS: up/down regulated expression of between 5 and -5-fold, or insignificant alterations

Gene Symbol	NCBI RefSeq ID	Fold change											
		**C vs. P1**	**C vs. P2**	**W1 vs. PR**	**C vs. C1**	**C vs. C2**	**W1 vs. CR**	**C vs. D1**	**C vs. D2**	**W1 vs. DR**	**C vs. T1**	**C vs. T2**	**W1 vs. TR**
*ABCB1*	NM_000927	**84,68**	**236,94**	**227,11**	N.S	N.S	N.S	**74,03**	**118,92**	**78,12**	N.S	N.S	N.S
*ABCB4*	NM_000443	**116,76**	N.S	N.S	N.S	N.S	N.S	**18,03**	**6,92**	**176,03**	N.S	N.S	N.S
*ABCC6*	NM_001079528	N.S	N.S	N.S	**-8,51**	**-7,08**	**-7,19**	N.S	N.S	N.S	N.S	N.S	N.S
*ABCG2*	NM_001257386	N.S	N.S	N.S	N.S	N.S	N.S	N.S	N.S	N.S	**204,17**	**167,94**	**258,73**
*BST2*	NM_004335	N.S	N.S	N.S	**-54,66**	**-12,69**	**-5,19**	N.S	**-21,27**	N.S	N.S	N.S	**13,57**
*EPHA7*	NM_004440	**8,14**	**14,58**	**8,13**	N.S	N.S	N.S	N.S	**5,53**	N.S	N.S	N.S	**5,37**
*ERAP2*	NM_001130140	N.S	N.S	N.S	**-5,98**	**-5,29**	**-10,83**	N.S	N.S	N.S	N.S	N.S	N.S
*HERC5*	NM_016323	N.S	N.S	N.S	N.S	N.S	N.S	N.S	N.S	N.S	**9,84**	**14,00**	**35,94**
*IFI16*	NM_001206567	**405,21**	N.S	**-6,47**	N.S	N.S	**-6,10**	**207,14**	**13,30**	**6,45**	N.S	N.S	N.S
*IFIH1*	NM_022168	**6,12**	N.S	N.S	N.S	N.S	N.S	**47,03**	N.S	N.S	**6,93**	**6,79**	**14,32**
*LIPG*	NM_006033	**-5,63**	**-10,46**	**-11,51**	N.S	N.S	N.S	N.S	N.S	N.S	N.S	N.S	**-9,18**
*MCTP1*	NM_001002796	**-19,18**	**-20,95**	**-23,26**	**-20,44**	**-13,56**	**-14,79**	N.S	**-19,29**	N.S	**-19,15**	**-21,37**	**-8,21**
*MYOT*	NM_001135940	**9,53**	N.S	**7,39**	N.S	N.S	N.S	N.S	N.S	N.S	**9,19**	**7,82**	**16,47**
*NSBP1*	AF250329	**-6,27**	**-5,10**	**-10,34**	N.S	N.S	**-13,92**	N.S	N.S	N.S	N.S	N.S	**-9,51**
*PCDH9*	NM_020403	**-6,34**	**-38,72**	**-5,31**	**12,73**	N.S	N.S	**7,74**	**-25,66**	**-8,99**	N.S	N.S	N.S
*PTPRK*	NM_001135648	**-15,10**	**-59,18**	**-38,18**	**-38,67**	**-96,85**	N.S	N.S	N.S	N.S	**-5,36**	**-7,07**	**-16,81**
*RUNDC3B*	NM_001134405	**13,07**	**56,57**	**10,26**	N.S	N.S	N.S	**8,56**	N.S	**17,55**	N.S	N.S	N.S
*S100A3*	NM_002960	N.S	N.S	N.S	**6,27**	**11,13**	N.S	N.S	N.S	N.S	**17,63**	**43,38**	**6,53**
*SAMD4A*	NM_001161576	**6,11**	**6,79**	N.S	N.S	N.S	N.S	N.S	N.S	N.S	**6,79**	**10,00**	**5,51**
*SEMA3A*	NM_006080	**-10,55**	**-21,08**	**-60,31**	N.S	N.S	N.S	**5,87**	N.S	N.S	N.S	N.S	N.S
*SPP1*	NM_000582	**19,42**	N.S	N.S	N.S	**54,95**	N.S	**13,75**	**13,06**	N.S	**213,44**	**229,77**	**279,28**
*TGFBI*	NM_000660	N.S	N.S	N.S	N.S	N.S	N.S	N.S	N.S	N.S	**27,95**	**17,48**	**11,10**

Collective, the expression levels of 22 genes were changed in three cell lines resistant to the same cytostatic drug. The expression of 13 genes was upregulated and that of nine genes was downregulated. We did not observe the overexpression of any gene in the cell lines resistant to CisPt. In contrast, we observed the underexpression of four genes in the cell lines resistant to this drug, which were the ABCC6, BST2, ERAP2 and MCTP1. In the Pac-resistant cell lines, we observed upregulated expression of three genes, ABCB1, EPHA7 and RUNDC3B. Six genes were underexpressed in the Pac-resistant cell lines LIPG, MCTP1, NSBP1, PCDH9, PTPRK and SEMA3A. The Dox-resistant cell lines were characterized by the increased expression of three genes, ABCB1, ABCB4 and IFI16. No gene was underexpressed in all three of the Dox-resistant cell lines. We observed the upregulated expression of eight genes in the Top-resistant cell lines ABCG2, HERC5, IFIH1, MYOT, S100A3, SAMD4A, SPP1 and TGFBI. The expression of two genes, MCTP1 and PTPRK, were downregulated in all three of the Top-resistant cell lines.

The expression level of some genes had changed in response to more than one cytostatic drug. The expression of ABCB1 was increased in all of the Pac- and Dox-resistant cell lines. The expression of EPHA7 was increased in three Pac-resistant cell lines and in one Dox- and one Top-resistant cell line. IFI16 expression was increased in all of the Dox-resistant cell lines but was also increased more than four-hundred-fold in the A2780PR1 Pac-resistant cell line. We observed the increased expression of IFIH1 in three Top-resistant cell lines and in the A2780DR1 and A2780PR1 cell lines that were resistant to Dox and Pac, respectively. MYOT expression was increased in three Top-resistant cell lines and two Pac-resistant cell lines. RUNDC3B expression was increased not only in all of the Pac-resistant cell lines but also in two of the Dox-resistant cell lines. S100A3 expression was increased in all of the Top-resistant cell lines and in two of the CisPt-resistant cell lines. SAMD4A expression was increased in three Top- and two Pac-resistant cell lines. SPP1 expression was very significantly increased in all of the Top-resistant cell lines and one CisPt-resistant cell line and was less significantly increased in two Dox-resistant cell lines and one Pac-resistant cell line. The level of expression of MCTP1 was decreased in all of the CisPt-, Pac- and Top-resistant cell lines and also in one Dox-resistant cell line. The expression of PTPRK was decreased in all of the Pac- and Top-resistant cell lines and in two of the three CisPt-resistant cell lines. The expression of PCDH9 was downregulated in three Pac-resistant cell lines, two Dox-resistant cell lines and in one CisPt- and one Top-resistant cell line.

Of the 22 genes for which the expression levels were analysed, the expression of three genes, ABCB1, ABCG2 and SPP1, was very significantly upregulated – by more than 50-fold – in three cell lines that were resistant to the same cytostatic drug. We also observed very greatly increased expression – by more 50-fold – of ABCB4, IFI16 and RUNDC3B, although this expression pattern was not shared by all of the cell lines resistant to the same cytostatic drug. The genes with the most highly downregulated expression – by more than 50-fold – were BST2, PTPRK and SEMA3A.

### Immunohistochemical analysis

Immunohistochemical analysis of selected (EPHA7, IFI-16, SPP1 and TGFBI) proteins was tested in ovarian cancer patients. The aim of this study was to verify whether the altered expression of analyzed genes and proteins observed in cell lines can be confirmed in a real cancer patient tissues as well.

**Figure 3:**
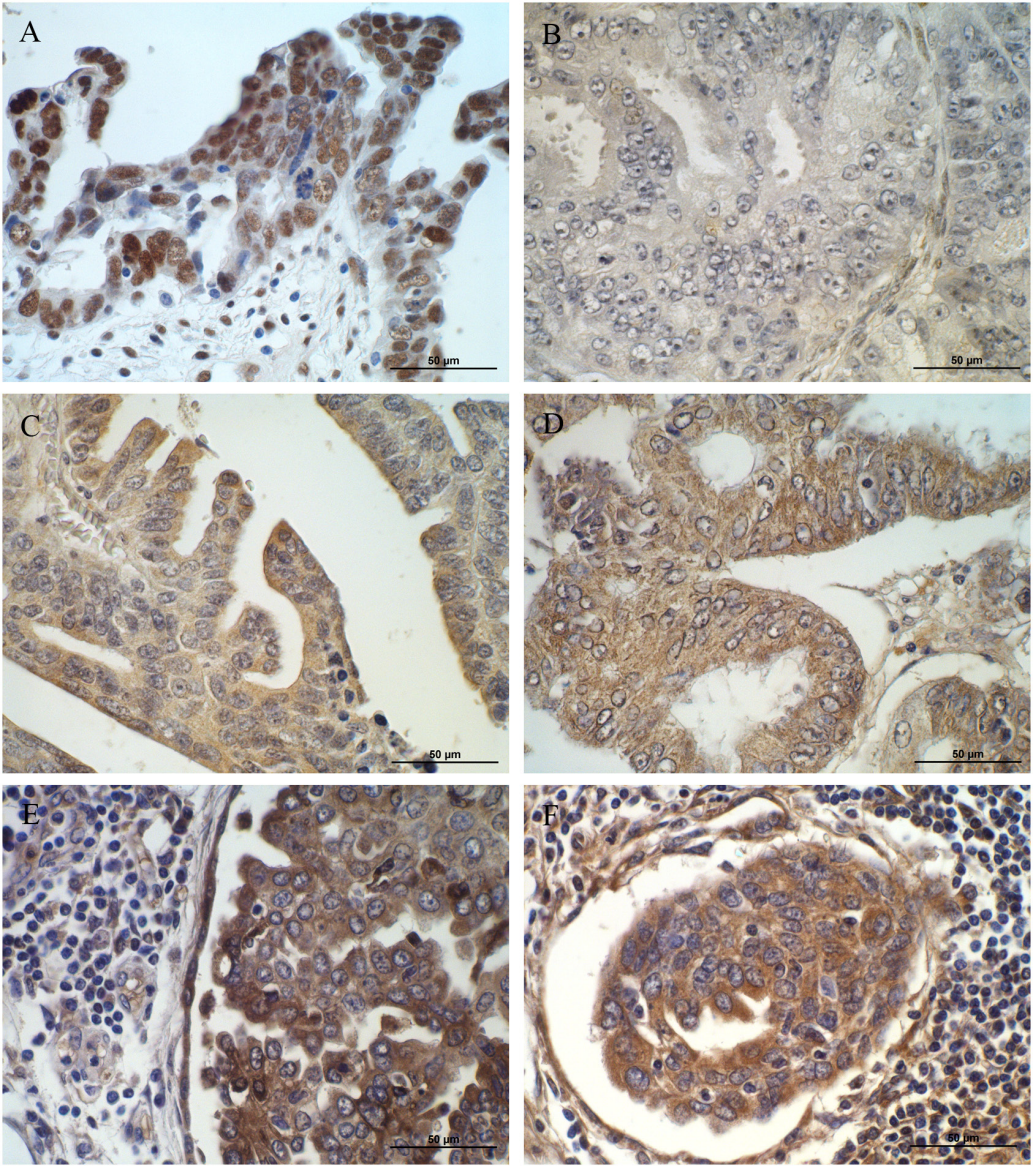
Immunohistochemical expression of (**A**) IFI16 in cancer and stromal cells of patient with ovarian serous adenocarcinoma (strong nuclear reaction); (**B**) IFI16 in ovarian endometrioid adenocarcinoma patient (no reaction); (**C**) EPHA7 in cancer cells of patient with ovarian serous adenocarcinoma (moderate cytoplasmic reaction); (D) EPHA7 in cancer cells of patients with endometrioid adenocarcinoma (moderate cytoplasmic reaction); (**E**) SPP1 in cancer and stromal cells of patient with ovarian serous adenocarcinoma (moderate cytoplasmic reaction); (**F**) TGFBI in cancer, stromal and infiltrating cells of patient with ovarian serous adenocarcinoma (moderate cytoplasmic reaction). Sections counterstained with hematoxylin. Scale bar = 50μm

The positive expression of IFI16 was observed in ovarian serous adenocarcinoma patients but not in ovarian endometrioid adenocarcinoma ones (Figure 3A, 3B). Strong (3) reaction was observed in nuclei of serous adenocarcinoma cancer cells. The immunopositive cells were detected also in the stromal cells surrounding the tumour.

EPHA7 protein was detected in both ovarian serous adenocarcinoma and endometrioid adenocarcinoma cancer cells (Figure 3C, 3D). In both types a weak (1) to moderate (2) reaction was observed in the cytoplasm of cancer cells.

Moderate (2) positive reaction was observed for SPP1 and TGFBI proteins (Figure 3E, 3F). Both proteins were mainly localized in the cytoplasm of ovarian serous adenocarcinoma cancer cells. The immunopositive cells were detected also in the stromal cells and inflammatory infiltrates surrounding the tumour.

## DISCUSSION

In this study alterations in the level of gene expression were found in ovarian cancer cell lines that were resistant to cytostatic drugs of first and second lines of chemotherapy. The genes with expression levels that were between 5- and 0.2-fold that of the control cells were considered not to be significantly affected, and the relationship between their expression levels and drug resistance will not be discussed.

The microarray data were confirmed by the qPCR results for genes representative of the MDR phenotype (MDR1 and MDR3) (data not shown). Additionally, the results of western blotting analysis of P-gp and BCRP protein expression also correlated with the alterations in the expression levels of the genes encoding these proteins (data not shown).

To our knowledge, the results presented here are the first to shown changes in the expression of genes in ovarian cancer cell lines that were resistant to cytostatic drugs of the first and second lines of chemotherapy. The levels of gene expression were determined in three resistant cell lines to make the results more credible. Our results indicated that new genes are related to the resistance to chemotherapeutic drugs.

First, we selected the genes that were overexpressed/underexpressed by at least 5-fold in the drug-resistant cell lines relative to the levels in the control cells (Figure 1). To obtain information about changes in gene expression that occurred in response to exposure to cytostatic drugs. Not surprisingly, we observed much greater similarity in the altered gene expression patterns of the R1 and R2 cell lines derived from the A2780 cell line that were resistant to the same cytostatic drug than between those of these two cell lines and that of a cell line resistant to the same cytostatic drug but derived from the W1 cell line. However, although the R1 and R2 cell lines were derived from the same A2780 cell line under the same cell-culture conditions, the general changes in their gene expression patterns differed. This result indicated that even in the same cell line and under identical cell culture conditions, the response to cytostatic drugs can be different. This finding indicate how difficult it is to predict the changes in gene expression that will occur in response to cytostatic-drug treatment. However, changes in the expression levels of some genes were common to all three cell lines that were the cellular response to cytostatic drugs and were therefore selected for more detailed analysis.

CisPt is the most important drug used to treat ovarian cancer [8] as well as many other types of cancer [12, 16]. We did not observe the increased expression of any gene in any of the cell lines resistant to CisPt. This finding is contrary to that of other study, which showed the overexpression of the drug transporter MRP2 [13], glutathione [14] or metallothioneins [15] in cell lines that were resistant to CisPt. These discrepancies may be due to the use of different models of study. In the other study, the investigators compared pairs of sensitive or resistant cell lines. In our study, we analyzed the expression of only the genes with altered expression levels in all of the cell lines that were resistant to the same cytostatic drug. Thus, genes that might be related to drug resistance but did not have altered expression levels in all cases were eliminated from our study and are not discussed here. The expression of four genes, ABCC6, BST2, ERAP2 and MCTP1, was downregulated in the CisPt-resistant cell lines.

ABCC6 is drug transporter from the ABC family that transports glutathione S-conjugated leukotriene. The role of ABCC6 in drug resistance is not well established. Increased expression of ABCC6 has been reported to impart a low level of resistance to etoposide, teniposide, doxorubicin, and daunorubicin [31]. The role of ABCC6 in CisPt resistance has not been reported. Nonetheless, the level of this drug transporter is suspected to be increased rather than decreased in drug-resistant cell lines.

Bone-marrow stromal antigen 2 (BST2), also known as a tetherin, is a protein that is associated with lipid rafts and is expressed mainly on B cells [32]. Expression of the BST2 gene was upregulated in tamoxifen-resistant MCF-7 human breast-cancer cells [33], human mammary xenografts resistant to tamoxifen [34] and in breast cancer patients with bone-marrow metastases [35]. However, the role of this gene in cytostatic-drug resistance is currently unknown. We observed downregulated expression of this gene in all of CisPt-resistant cell lines, with very strong downregulation in the A2780CR1 cell line and strong downregulation in the A2780DR2 cell line. However, the expression level of this gene in the W1TR cell line was increased. Downregulated expression of this gene in all of the CisPt-resistant cell lines suggested that it might be a marker of CisPt resistance.

ERAP2 (endoplasmic reticulum aminopeptidase 2) plays a central role in peptide trimming, a process required for the generation of most HLA class I-binding peptides [36]. To date, this protein has not been reported to play a role in cytostatic-drug resistance. However, it has been reported that the inhibition of ER-peptide trimming play a key role in stimulating innate and adaptive anti-tumour immune responses [37]. We observed downregulated ERAP2 expression in the CisPt-resistant cell lines, which suggested that CisPt-resistant cells might be more susceptible to immune-response components than other cells.

The expression of the MCTP1 (multiple C2 domains, transmembrane 1) gene was strongly downregulated in all of the CisPt-, Pac- and Top-resistant cell lines, as well as in one of the Dox-resistant cell line. This result suggested that downregulation of MCTP1 expression might be an unspecific response of cancer cells to cytostatic-drug treatment. This protein is poorly described in the literature. C2 domains are primarily found in signal-transduction proteins and membrane-trafficking proteins. In both of these types of proteins, the C2 domains are responsible for regulating their functions through Ca^2+^ binding [38].

The second drug used in first line ovarian-cancer chemotherapy is Pac. Resistance to this drug is mainly related to the MDR phenotype of cancer cells and to the level of MDR1 (ABCB1) expression. We previously reported increased expression of the ABCB1 gene in Pac- and Dox-resistant cell lines and discussed the implications of this finding [39, 40].

A new gene that was found to be expressed in the Pac-resistant cell lines is EPHA7. The EPHA7 (EPH receptor A7) gene encodes the ephrin receptor, which belongs to the protein-tyrosine kinase family and functions as receptor-tyrosine kinase that binds ephrin-A-family ligands. The ligand of this receptor is EFNA5. Forward signalling via EPH receptor A7 may result in the activation of components of the ERK signalling pathway [41]. To our knowledge it is the first time where elevated expression of EPHA7 is observed in ovarian cancer patients. However expression of EPHA7 was observed in other cancers. The expression of EPHA7 is downregulated in colorectal cancer [42] and upregulated in prostate [43] and lung cancer [44]. In gallbladder adenocarcinoma, the expression level of EPHA7 was an independent poor-prognostic predictor, and the elevated expression of EPHA7 was closely related to carcinogenesis, disease progression and a poor prognosis [45]. In hepatocellular carcinoma, a high level of expression of EPHA7 protein may play an important role in malignant transformation and tumour progression, invasion and metastasis [46]. In summary, elevated expression of EPHA7 is a poor prognostic factor for many cancers and is correlated with cancer progression and metastasis. To our knowledge, this study is the first to show that EPHA7 might play a role in Pac resistance and development of ovarian cancer.

The RUNDC3B (RPIP9 - Rap2-Interacting Protein 9) gene encodes a poorly characterized protein that interacts with Rap2 and belongs to the Ras superfamily of GTPases. It was reported that RPIP9 was activated in a high proportion of breast carcinomas and that this occurrence was significantly correlated with their metastasis to lymph nodes. Interestingly, the sequences of the RPIP9 and MDR1 genes overlap on chromosome 7 [47]. It is possible that RPIP9 is expressed in Pac-resistant cell lines due to the activation of MDR1 in these cells. Similarly, RPIP9 was also expressed in two of the three Dox-resistant cell lines that also expressed MDR1. It is also possible that by interacting with RAP2, RPIP9 plays a role in signal transduction leading to a more invasive and drug-resistant phenotype.

Downregulation of the expression of certain genes is related to specific cellular phenotypes. In the Pac-resistant cell lines, we observed downregulated expression of the LIPG gene. LIPG is an endothelial lipase that is involved in the hydrolysis of high-density lipoproteins (HDL) [48]. We did not found any data in the literature concerning the expression of LIPG in drug-resistant cancers or cancer cell lines. It is possible that Pac treatment led to the downregulation of LIPG expression.

Another gene with downregulated expression in three Pac-resistant cell lines was NSBP1, which encodes a chromatin- and nucleosome-binding protein that preferentially binds to and unfolds euchromatin via a Nucleosome Binding Domain (NBD) and thus modulates transcription [49]. This protein has been reported to induce the differentiation of mouse embryonic stem cells [50]. This gene was observed to be overexpressed in squamous cell carcinoma [51] and prostate cancer [52] in humans, as well as in the highly metastatic MDA-MB-435HM breast cancer cell line [53]. The elevated expression of NSBP1 in this cell line suggests that it plays a role in tumourigenesis. In contrast, in this study, we observed downregulated expression of this gene in Pac-resistant cell lines, suggesting that they present less metastatic potential than parental cell lines.

PCDH9 (protocadherin 9) is a transmembrane protein containing cadherin domains that is most likely involved in calcium dependent cell adhesion and is involved in signalling at neuronal synaptic junctions [54]. PCDH9 has been reported to be a tumour-suppressor gene in human gliomas. The loss of PCDH9 expression was associated with a higher histological grade of tumour and significantly shorter survival times [55]. In hepatocellular carcinoma (HCC) cells, the loss of PCDH9 expression facilitated tumour-cell migration and epithelial-mesenchymal transition (EMT) [56]. Thus, the expression of PCDH9 is downregulated in tumours and the loss of its expression can be related to a more invasive phenotype.

The expression of the PTPRK (Protein tyrosine-phosphatase, receptor-type kappa) gene was downregulated in all of the Pac- and Top-resistant cell lines, as well as in two CisPt-resistant cell lines, with very strong downregulation observed in the A2780PR2 and A2780CR2 cell lines (by more than 50-fold). The PTPRK gene is located in a putative tumour-suppressor region of the human genome, on the long arm of chromosome 6 [57]. This gene was one of the most downregulated observed in our study, suggesting its significant role in the development of cytostatic-drug resistance. It has been reported that PTPRK expression is downregulated in many cancer types and cancer-cell lines including, melanoma [58], lung cancer [59], prostate cancer [60] and breast cancer [61]. Breast cancer patients with decreased PTPRK transcript levels have shorter survival times and a higher probability of metastases [61]. The role of this gene in ovarian cancer was not reported. Our results suggest that its reduced expression is associated with the development of drug resistance in ovarian cancer. The loss of active PTPRK in gliomas was associated with a higher level of resistance to chemotherapy and reconstitution of wild-type PTPRK in malignant glioma-cell lines improved the effect of conventional therapeutics [62]. Decreased levels of PTPRK expression or the loss of PTPRK activity due to mutation led to increased tyrosine-phosphorylation-based signalling, which is a major driving force in the development and progression of tumours. Thus, the decreased PTPRK expression in our cell lines might lead to increase the expression levels of genes involved in drug resistance.

SEMA3A (collapsin-1) is a secreted protein that is a member of semaphorin family. Normally, this protein activates signal transduction in neuronal cells through its receptor NP1/PlexA [63]. We observed the SEMA3A gene downergulation in the Pac-resistant cell lines, with more than 60-fold downregulation found in the W1PR cell line. These results are consistent with the results of another study. Downregulated SEMA3A expression was also observed in non-small cell lung cancer (NSCLC) [64]. It was suggested that lower levels of SEMA3A may promote pleural and vascular invasion and lymph-node metastasis. Decreased expression of SEMA3A in gastric carcinomas was associated with poor differentiation and with invasion and metastasis [65].

The expression of ABCB1 and ABCB4 in Dox-resistant cell lines was associated with the MDR phenotype, as we previously reported [20, 21, 39, 40].

The expression level of the IFI16 (interferon, gamma-inducible protein 16) gene was increased in the Dox-resistant cell lines and in the A2780PR1 cell line, which was resistant to Pac. We observed a greater than four-hundred-fold increase in the expression level of this gene in the A2780PR1 cell line, suggesting its role in resistance to cytostatic drugs. IFI16 interacts with p53 and retinoblastoma protein and inhibits cellular growth via the Ras/Raf signalling pathway [66]. Contradictory data regarding the role of IFI16 in cancer has been reported in the literature. It has been reported that most breast-cancer cell lines have a decreased level of IFI16 mRNA compared with that in normal epithelial cells [67]. Similar observations were made regarding prostate cancer [68]. The authors suggested that the loss of IFI16 expression contributed to the development of breast or prostate cancer. In contrast, in ovarian cancer cells, IFI16 appeared to be involved in drug resistance. The expression levels of genes in primary epithelial ovarian cancer tissues that were sensitive or resistant to chemotherapeutics were compared. One of genes that was overexpressed in drug-resistant tissues was IFI16 [69]. Our results also indicate that protein expression of IFI16 could differ in histologically different types of ovarian cancer. This finding is consistent with our cell culture results showing the overexpression of IFI16 in drug-resistant cell lines. Thus, the role of IFI16 in cancers may be cancer-type specific; however, in ovarian cancer cells, its overexpression appears to be related to the drug-resistant phenotype.

Top, a topoisomerase I-poison is a cytostatic drug used in the second-line chemotherapy of ovarian cancer. The main mechanism underlying Top resistance includes its efflux by the drug transporter ABCG2 and mutations in DNA topoisomerase I [28]. We previously reported increased expression of ABCG2 in the investigated cell lines and discussed the implications [20, 39, 40]. However, in this study we observed the increased expression of eight additional genes that appeared to be related to Top resistance.

HERC5 is an E3 ubiquitin-protein ligase [70] that is a positive regulator of the innate antiviral response in cells treated using interferon [71]. It has been reported that the level of HERC5 is a prognostic marker in lung cancer [72]; however, its role in drug resistance is unknown.

Three other genes, IFIH1, MYOT and SAMD4A, that were found to be overexpressed in the Top-resistant cell lines were not previously reported to be related to drug resistance or even to any cancer. The IFIH1 gene encodes the MDA5 (Melanoma Differentiation-Associated protein 5) protein, which recognizes dsRNA and participates in the antiviral response [73]. The MYOT gene encodes myotylin, a skeletal muscle protein that is found within the Z-discs of sarcomeres and is involved in the regulation of myofibril assembly and stability [74]. In human tissues, myotylin expression is largely restricted to striated muscles and nerves. SAMD4A (Sterile Alpha Motif Domain Containing 4A) is a translational repressor of genes encoding SRE-containing messenger proteins [75]. In the study, we showed that these genes are expressed in all of the Top-resistant cell lines. This finding suggested that IFIH1, MYOT and SAMD4A are involved in the resistance to this drug.

S100A3 is a calcium binding protein of unknown function. Other members of the S100 proteins family are involved in regulating cell-cycle and cell differentiation [76]. The expression level of S100A3 was increased in human colorectal cancer cells compared that in normal control cells [77]. In human castration-resistant prostate cancers, the expression level of S100A3 was increased and inhibiting its expression resulted in the suppression of tumour growth [78]. These data suggest that our Top-resistant cell lines might have an increased tumorigenic potential compared with the parental cell line.

SPP1 (secreted phosphoprotein 1), also known as osteopontin (OPN), is a secreted protein that is expressed in bone tissue [79]. Its expression has also been reported in many cancers including ovarian cancer [80] and it appears to be involved in drug resistance and in the progression and metastasis of tumours [81]. Our results confirm expression of OPN in ovarian cancer and suggest its role in Top-resistance. The role of OPN in the resistance to CisPt [82, 83] and to P-gp substrates [83, 84], has been reported. In a very elegant study, Das and coworkers [83] showed that OPN treatment increased the levels of resistance to CisPt, Dox and Pac and induced the expression of ABCB1 and ABCG2. In the present study, we observe the overexpression of OPN in all of the Top-resistant cell lines. All of these cell lines also expressed ABCG2 at a very high level. Thus, OPN appeared to induce ABCG2 expression in the Top-resistant cell lines and by doing so, may be responsible for Top resistance. In similar manner, OPN expression might also be responsible for the P-gp expression and Dox and Pac resistance in our cell lines. OPN overexpression might induce CisPt resistance in the A2780CR2 cell line via a different mechanism. In small-cell lung cancer, OPN induced CisPt resistance by blocking caspase-9- and caspase-3-dependent apoptosis [82]. In summary, OPN appeared to be involved in drug resistance mainly through stimulating ABC-transporter expression.

We previously reported that TGFBI (transforming growth factor-beta-induced protein) was expressed in Top-resistant cell lines in the context of the expression of extracellular matrix (ECM) components [85, 86]. In this study TGFBI expression appeared to be a marker of Top resistance. To our knowledge, the role of this protein in Top resistance is unknown. However, we found much data concerning the expression of TGFBI in ovarian cancer and in Pac-resistant cells. Nearly all of the reports indicated that the expression level of TGFBI in ovarian cancers [87] and ovarian cancer cell lines [88] was decreased via promoter methylation. Furthermore, decreased levels of TGFBI expression have been correlated with resistance to Pac [88, 89]. However, the results of other studies indicated that TGFBI promoted the metastatic potential of ovarian cancer cells by promoting their motility, invasion, and adhesion to peritoneal cells [90] and that overall survival (OS) was significantly shorter in serous-epithelial ovarian cancer patients whose tumours expressed TGFBI [91]. Our results also indicate that TGFBI is expressed in ovarian cancer patients. In view of these conflicting data, it is difficult to explain the role of TGFBI in ovarian cancer. However, because TGFBI was expressed in all of the Top-resistant ovarian cancer cell lines, its expression might be a marker of Top-resistance in ovarian cancer.

## MATERIALS AND METHODS

### Reagents and antibodies

Cisplatin, doxorubicin, topotecan, and paclitaxel were obtained from Sigma (St. Louis, MO, USA). TRIzol reagent, RPMI-1640 medium, foetal bovine serum, penicillin, streptomycin, amphotericin B (25 μg/ml) and L-glutamine were also purchased from Sigma (St. Louis, MO, USA). A Cell Proliferation Kit I (MTT) was purchased from Roche Diagnostics GmbH (Mannheim, Germany). An Affymetrix GeneChip® 3 'IVT Express Kit (Affymetrix, Santa Clara, CA, USA) and Affymetrix GeneChip Human Genome U219 microarrays were purchased (Affymetrix, Santa Clara, CA, USA) were utilized. Mouse monoclonal anti-EPHA7 antibody and mouse monoclonal anti-IFI16 antibody were purchased from Abnova (Taipei, Taiwan). Rabbit polyclonal anti-SPP1 antibody was purchased from Proteintech (Chicago, USA). Rabbit polyclonal anti-TGFBI antibody was purchased from Atlas Antibodies (Bromma, Sweden).

### Cell lines and cell culture

In this study, we used two ovarian cancer cell lines, the established ovarian cancer A2780 cell line and the primary ovarian cancer W1 cell line.

The human ovarian carcinoma A2780 cell line was purchased from ATCC. A2780 sublines that were resistant to CisPt [A2780CR1 and A2780CR2 (A2780 cisplatin resistant)], Pac [A2780PR1 and A2780PR2 (A2780 paclitaxel resistant)], Dox [A2780DR1 and A2780DR2 (A2780 doxorubicin resistant)] and Top [A2780TR1 and A2780TR2 (A2780 topotecan resistant)] were generated by exposing A2780 cells to the relevant drugs at incrementally increased concentrations.

The human ovarian cancer W1 cell line was established using ovarian cancer tissue obtained from an untreated patient. W1 sublines resistant to CisPt [W1CR (W1 cisplatin resistant)], Dox [W1DR (W1 doxorubicin resistant)], Top [W1TR (W1 topotecan resistant)] and Pac [W1PR (W1 paclitaxel resistant)] were obtained by exposing W1 cells to the drugs at incrementally increased concentrations.

All resistant cell lines were generated in our laboratory. The cells were seed in the concentration of 10 thousand cells/cm^2^ in 25 cm^2^ flask in dedicated media supplemented with appropriate drug. The established concentrations of the initial drugs exposure were of: CisPt 20 ng/mL, Dox 10 ng/mL, Top 0,5 ng/mL, and Pac 1 ng/mL. Each cell line was exposed three times for 3-day periods during a 3–6-week period allowing for growth recovery between cycles. The drug dose was doubled after the completion of three cycles and the procedure was repeated until the final drug levels were achieved.

The final concentrations used for selection were 1000 ng/ml Cis, 1100 ng/ml Pac, 100 ng/ml Dox, and 24 ng/ml Top. These concentrations were chosen based on the results of Dietel et al., 1997 [29] and were two-fold higher than the plasma concentrations of the respective drugs 2 hours after intravenous administration.

All of the cell lines were maintained as monolayers in complete medium [MEM medium (A2780) or RPMI-1640 (W1) supplemented with 10% (v/v) foetal bovine serum, 2 pM L-glutamine, penicillin (100 units/ml), streptomycin (100 units/ml) and amphotericin B (25 μg/ml)] at 37°C in a 5% CO_2_ atmosphere.

### RNA isolation and preparation of microarray an RQ-PCR reactions

The RNA was isolated from the A2780, W1 cells and all of the resistant cells using TRIzol reagent, according to the manufacturer's protocol. The RNA was quantified using spectrophotometry by measuring the absorbance values at 260 nm and 280 nm, and the 260/280 nm ratio was used to estimate the level of protein contamination. The 260/280 nm ratios of the samples ranged from 1.8 to 2.0. The extent of RNA degradation was evaluated using an electrophoretic method employing a 1% denaturing agarose gel and by estimating the RIN factor using a Bioanalyzer 2100 system (Agilent Technologies, Inc., Santa Clara, CA, USA). RIN values ranged from 8.5 to 10, with an average value of 9.2. Additionally, each sample was diluted to a final working concentration of 100 ng/μl. All of the samples were prepared in triplicate. The cDNA for the microarray analysis was synthetized in two steps (separate synthesis for first and second strand) using an Affymetrix GeneChip® 3′ 'IVT Express Kit and 100 ng/μl of RNA, according to the Affymetrix GeneAtlas 3′ IVT Express Kit protocol. The next steps, which were *in vitro* transcription (resulting in cRNA populations), biotin labelling, and cRNA fragmentation were performed using the same protocol.

### Microarray hybridization and scanning

The samples were loaded onto and hybridized to Affymetrix GeneChip Human Genome U219 microarrays, together with control cRNA and oligo B2. Hybridization was conducted at 45°C for 16 hours. using an AccuBlock™ Digital Dry Bath (Labnet International, Inc. NY, USA) hybridization oven Next, the microarrays were washed and stained according to manufacturer's protocol using an Affymetrix GeneAtlas™ Fluidics Station (Affymetrix, Santa Clara, CA, USA), and the chips were scanned using an Affymetrix GeneAtlas™ Imaging Station (Affymetrix, Santa Clara, CA, USA). The scans of the microarrays were saved as *.CEL files on hard disks.

### Analysis of the microarray results and gene screening

Quality control (QC) studies were performed using Affymetrix GeneAtlas^™^ (Affymetrix, Santa Clara, CA, USA) software, according to the manufacturer's standards. Secondary quality control studies were performed using Partek® Express^™^ Software (Partek, Inc., Chesterfield, MO, USA). Gene-screening analysis of the QC-based results and statistical analysis (non-parametric Mann-Whitney test using α=0.05) were performed using the same software. As a result, a table showing the most significant fold changes in the levels of gene expression of the resistant cells relative to those of the parental cells was developed, after which it was imported into the Pathway Studio® Explore platform (Ariadne Genomics, Rockville, MD, USA), in which pathway studies was performed. The genes with greatest fold changes in expression between the drug-resistant and parental cell lines were listed.

**Figure 4:**
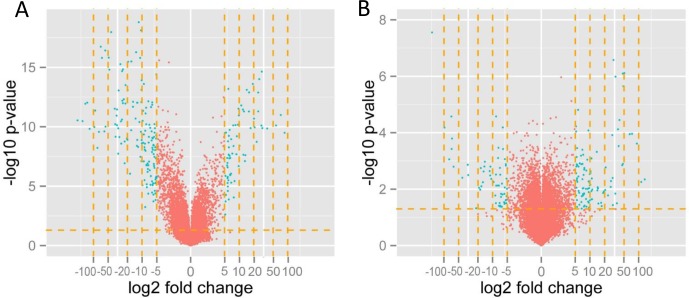
Volcano plot displaying the genes with expression levels that were up/downregulated by fivefold and more (green dots) in A2780TR1 cells with respect to the levels in sensitive A2780 cells **(A)** and W1DR with respect to sensitive W1 **(B)**. Volcano plotting filtered the genes with a fold change in expression levels of between 0.2- and 5-fold (red dots).

To visualize the effect of filtering the data, we applied the gene list to volcano plotting using a five-fold change in the expression level as the threshold (genes with expression upregulated more than 5-fold and downregulated -5-fold) (Figure 4). threshold was applied in preparing the gene table and in final analytical step, the genes related to cytostatic drug-resistance were selected. The volcano plots and the list of genes were created using the R language-based (http://www.r-project.org, version 2.14) Bioconductor (http://www.bioconductor.org) package.

### Immunohistochemistry

Immunohistochemical analysis was performed on transverse 5 μm formalin-fixed and paraffin embedded sections from human ovarian carcinoma placed on the SuperFrost/Plus microscope slides. We have investigated tissues from patients with ovarian serous adenocarcinoma and ovarian endometrioid adenocarcinoma. The analysis of EPHA7, IFI16, SPP1 and TGFBI expression was performed by use of the polymer-based immunohistochemical (IHC) technique [30] The primary antibodies used as follows: EPHA7 (1:300, mouse monoclonal anti-EPHA7 antibody, Abnova, Taipei, Taiwan), IFI16 (1:200, mouse monoclonal anti-IFI16 antibody, Abnova, Taipei, Taiwan), SPP1 (1:100, rabbit polyclonal anti-SPP1 antibody, Proteintech, Chicago, USA), TGFBI (1:100, rabbit polyclonal anti-TGFBI antibody, Atlas Antibodies, Bromma, Sweden).

The slides were dewaxed with xylene, and gradually hydrated. Activity of endogenous peroxidase was blocked by 30 minute exposure to 1% H_2_O_2_. The sections were incubated with appropriate primary antibodies overnight at 4°C and followed by incubation with EnVision Detection System Peroxidase/DAB, Rabbit/Mouse for 30 minutes (Dako REALTMEnVisionTM Detection System peroxidase/DAB+, Rabbit/Mouse, Dako, Glostrup, Denmark). The sections were then counterstained with hematoxylin, dehydrated and mounted.

Histological slides with protein expression were examined under an optical Olympus BH-2 microscope coupled to a digital camera. Color microscope images were recorded using LUCIA Image 5.0 computer software (Nikon, Tokyo, Japan).

The expression of given markers (only clearly labelled cells with cytoplasmic/nuclear signal were considered), was calculated taking into account the mean proportion of immunopositive cancer cells among all cancer cells counted in 10 light microscope fields each at magnification of 400x (at least 100 cancer cells per one microscopic field). Expression was evaluated using the semi-quantitative scale in which the score of 0 (negative) corresponded to no staining observed or less than 10% of cancer cells weakly positive; score 1 (weak) corresponded to 11% to 50% positive cancer cells; the score 2 (moderate) corresponded to 51% to 75% positive cancer cells; score 3 (strong) corresponded to up to 75% positive cancer cells.

## CONCLUSIONS

In summary, the alteration of the expression levels of many genes was observed in ovarian cancer cells in response to treatment with first- and second-line chemotherapeutic cytostatic drugs. Approximately half of these genes were not previously associated with any cancer including ovarian cancer and and not described in term of cytostatic-drug resistance. Changes in expression levels of these genes in three ovarian cancer cell lines that were resistant to the same cytostatic drug indicated that these changes might be specific markers of deleterious cellular responses to chemotherapy. Expression of selected genes was confirmed at protein level in ovarian cancer patients. Thus, up/downregulation of the expression of these genes pre- versus post-chemotherapy could be predictive in term of the response to treatment. For confirmation of these hypotheses, gene-expression studies should be conducted using an animal model of ovarian cancer and using samples of primary and metastatic tumours taken from ovarian cancer patients before and after chemotherapy is administered.
